# Integration of scRNA-seq and bulk RNA-seq to reveal the association and potential molecular mechanisms of metabolic reprogramming regulated by lactylation and chemotherapy resistance in ovarian cancer

**DOI:** 10.3389/fimmu.2025.1513806

**Published:** 2025-02-28

**Authors:** Fang Ren, Xiaoao Pang, Feng Jin, Nannan Luan, Houhua Guo, Liancheng Zhu

**Affiliations:** Department of Obstetrics and Gynecology, Shengjing Hospital of China Medical University, Shenyang, China

**Keywords:** ovarian cancer, chemoresistance, lactylation, single cell sequencing, metabolic pathways, S100A4, ALDH1A1

## Abstract

**Objective:**

Ovarian cancer (OC) ranks among the foremost causes of mortality in gynecological malignancies, with chemoresistance being the primary factor contributing to unfavorable prognosis. This work seeks to clarify the mechanisms of resistance-related lactylation in OC, intending to offer novel theoretical foundations and therapy strategies for addressing chemoresistance.

**Methods:**

Through the combined analysis of bulk RNA-seq and single-cell RNA-seq data, we initially found lactylation genes linked to chemoresistance. Subsequently, we employed differential expression analysis, survival analysis, enrichment analysis, and other methodologies to further investigate the roles and molecular mechanisms of these genes in tumor resistance. Ultimately, we investigated the differential expression of these genes in resistant and non-resistant tissues and cells via experimentation.

**Results:**

We found two candidate genes associated with lactylation chemoresistance, ALDH1A1 and S100A4. Analysis of single-cell data indicated that tumor cells represent the primary cell subpopulation relevant to resistance studies. Subpopulation analysis indicated that several tumor cell subtypes were markedly linked to resistance, with elevated expression levels of ALDH1A1 and S100A4 in the resistant subpopulation, notably correlating with various immunological and metabolic pathways. Analysis of metabolic pathways indicated that oxidative phosphorylation and glycolysis activity was elevated in the resistant subpopulation, and lactic acid buildup was associated with chemoresistance. The investigation of the marker gene protein-protein interaction network in the resistant subgroup elucidated the intricate interactions among these genes. The expression levels of ALDH1A1 and S100A4 in the OC tissues of the platinum-resistant cohort were markedly elevated compared to the sensitive cohort, with a considerable rise in S100A4 expression observed in resistant OC cells, demonstrating co-localization with lactylation.

**Conclusion:**

This work elucidates the significant function of lactylation in OC chemoresistance and identifies ALDH1A1 and S100A4 as possible genes associated with drug resistance. These findings enhance our comprehension of the mechanisms behind chemoresistance in OC and offer critical insights for the formulation of novel therapeutic options.

## 1 Introduction

Ovarian cancer (OC) is the most fatal gynecologic malignancy, characterized by the greatest mortality rate, with an annual incidence of 314,000 cases and 207,000 deaths globally. Approximately 80% of OC patients receive diagnoses at advanced stages III and IV, with associated 5-year survival rates of only 27% and 13%, respectively (1). Epithelial OC constitutes about 85-90% of all instances, with initial and subsequent treatment resistance markedly influencing unfavorable outcome. Significant differences in energy metabolism are present between malignant and normal cells (1, 2).

Malignant cells typically alter their energy metabolism from oxidative phosphorylation to aerobic glycolysis, leading to increased glycolytic flux and heightened lactate production. The accumulation of lactic acid is linked to the promotion of tumor invasion, the induction of angiogenesis, and the occurrence of recurrence. Targeting the metabolic characteristics unique to tumor cells may represent a promising strategy for effective cancer treatment by disrupting glycolytic processes or altering lactate metabolism (3, 4).

Research indicates that chemoresistant OC cells demonstrate heightened lactate production and oxygen consumption, suggesting a greater accumulation of lactate in chemoresistant tissues and the formation of conditions favorable for lactylation (5). The suppression of glucose consumption and lactate production in OC results in antitumor activity through the inhibition of the Warburg effect (6, 7). The findings indicate that abnormal metabolic regulation linked to lactylation plays a role in drug resistance mechanisms, warranting additional research into the specific molecular processes involved. Lactylation is recognized as a prevalent mechanism contributing to drug resistance. The accumulation of lactate leads to lactic acidosis in tumor cells and facilitates metabolic reprogramming, thereby enhancing metastasis and chemoresistance in diverse tumors. Similar mechanisms may clarify the metabolic and transcriptional regulatory characteristics observed in chemoresistant OC, addressing existing research gaps and establishing a foundation for overcoming chemoresistance.

This study systematically investigates the mechanisms underlying platinum resistance in OC through the integration of single cell and bulk RNA sequencing data. We examined two lactylation-related genes (LacRGs), ALDH1A1 and S100A4, which may be linked to drug resistance and lactate metabolism. Our findings indicate a substantial presence of tumor cells in both resistant and non-resistant groups, underscoring the necessity of investigating tumor cells to comprehend drug resistance. The elevated lactate levels in tumor cells expressing ALDH1A1 and S100A4 from the resistant group, along with the increased expression of ALDH1A1, suggest that lactylation is integral to the development of drug resistance, resulting in a higher accumulation of lactate products in this group. The findings suggest that metabolic abnormalities associated with lactate contribute to chemoresistance and offer new theoretical insights for clinical decision-making.

## 2 Materials and methods

### 2.1 Downloading and preprocessing data

Bulk sequence transcriptome data: downloaded GSE26712 (8) and GSE15372 (9) transcriptome data from the GEO database, along with clinical information, as presented in **Table 1**. The GEO data underwent the following preprocessing steps: Probes corresponding to the gene were identified, and empty probes were eliminated. The median value of multiple probes associated with the same gene was selected as the gene’s expression level, which was subsequently utilized for differential expression analysis.

**Table 1:** Data collection.

Dataset ID	Data type	Platform	Sample number	Purpose
GSE26712	Bulk	GPL96	10/185 (tumor/normal)	Differential expression analysis
GSE15372	Bulk	GPL570	5/5 (resistance/non-resistance)	Differential expression analysis
RC47Y6M9MP.1	Single cell	Illumina	4/9 (resistance/non-resistance)	Single cell data analysis

Gene expression matrix data for single cell: the expression profiles of a single-cell dataset of OC were obtained from the Mendeley Data database (10) (https://data.mendeley.com/datasets/rc47y6m9mp/1), focusing on chemoresistant and non-resistant primary OC tissues. This project analyzed 13 samples, comprising 4 resistant and 9 non-resistant specimens.

A total of 323 LacRGs were identified based on previous study (11).

### 2.2 Differential and survival analysis for bulk sequence data

The R package limma (v3.56.2) was employed to analyze differences in LacRGs in the GSE26712 dataset. The criteria for differentially expressed genes (DEGs) were established as |log2FoldChange|>1 and p value < 0.05 (12). Kaplan-Meier survival analysis was conducted using GSE26712 data to identify prognostic LacRGs, focusing on genes exhibiting differential expression between normal and tumor. Secondly, the LacRGs in the GSE15372 dataset was analyzed to identify DEGs between chemoresistant and non-resistant groups. The intersection with prognostic LacRGs was determined to identify the potential lactylation resistance gene (Lat_Resi gene). Gene set enrichment analysis (GSEA) was conducted, integrating HALLMARK and KEGG gene sets.

### 2.3 Analysis of single-cell sequencing data

Quality control of the single-cell dataset samples was conducted utilizing the R package Seurat (v4.3.0.1). The threshold for excluding low-quality cells and low-expressed genes was established as follows: The feature count per cell ranged from 200 to 8000, while the count of observations per cell varied between 200 and 75000. The percentage of mitochondrial genes in each cell was below 10%. Secondly, the NormalizeData function was employed for normalization, while the FindVariableFeatures function (nfeatures=2000) was utilized to identify hypervariable genes. Batch correction among samples was conducted using the R package Harmony (v0.1.1) to mitigate the influence of batch effects on subsequent analyses. The data were subsequently scaled, linearly transformed via ScaleData, and subjected to dimensionality reduction through RunPCA. The elbow plot was generated using ElbowPlot to assess the dimensionality reduction of the data. Thirty principal components were chosen for subsequent analysis, utilizing the FindNeighbors and FindClusters functions for cell clustering (dims = 1:30, resolution = 2). Cell annotations from the original dataset authors and relevant reference (10) were utilized in this analysis to further differentiate annotated epithelial cells. Additionally, InferCNV (v1.16.0) was employed to compare the CNV levels of each epithelial cluster for the identification of tumor cells. The proportion of each cell subpopulation between the chemoresistant and non-resistant groups was compared, focusing on cell types that were up-regulated in the chemoresistant group.

### 2.4 Annotations of cell subgroups and association with prognosis

Initially, utilizing the single-cell data, the tumor cells were categorized into subgroups (resolution = 0.01), and the heatmap illustrating marker gene expression within each subgroup was presented. Marker genes of each tumor cell subset were analyzed using the criteria of adjusted p<0.05 and |log2FC|>0.25. Functional enrichment analysis of gene sets and pathway enrichment analysis were conducted utilizing Gene Ontology (GO) and Kyoto Encyclopedia of Genes and Genomes (KEGG). Secondly, the identification of potential chemoresistant subsets involves differentiating tumor cell subtypes based on the ratio of chemoresistant to non-chemoresistant cells. The subgroup representing over 80% of the drug resistance category was designated as the chemoresistant subgroup, while the subgroup comprising more than 80% of the non-resistance category was labeled as the non-resistant subgroup. The remaining groups were classified as mixed populations. The genes that were up-regulated and down-regulated in the chemoresistant subgroup compared to the non-resistant subgroup were analyzed, and an enrichment analysis of the DEGs between these two subgroups was conducted.

The IC50 values of cisplatin for each tumor cell were determined using the R package oncoPredict (v1.2), and the differences in IC50 values between resistant and non-resistant subgroups were analyzed.

According to the GSE26712 data, the scores of chemoresistant subgroups were calculated using the GSVA method, and samples were grouped based on the optimal threshold method. The Kaplan-Meier survival curve effectively identifies the proportion of chemoresistant subgroups and indicates poor tumor prognosis.

### 2.5 Analysis of chemoresistance mechanisms associated with lactylation and corresponding metabolic abnormalities

The ssGSEA enrichment scores for metabolic and immune pathways were calculated for each subgroup. The specific immune and metabolic pathways associated with the chemoresistant subgroup were identified through a comparison with the non-resistant subgroup. The correlation between the subset of Lat_Resi genes specific to the resistant subgroup and immune and metabolic pathways was assessed to identify the regulatory pathways significantly associated with lactate.

ScMetabolism was employed to quantify the metabolic scores of each tumor cell subpopulation, facilitating a comparison and analysis between the resistant and non-resistant subpopulations to identify significant metabolic pathways. scFEA was employed to quantify the metabolite abundance within each tumor cell subpopulation and to ascertain the enrichment of metabolic pathway products in these subpopulations. The relationship between metabolic levels and the IC50 of cisplatin was assessed using Spearman correlation. Cells were categorized into high and low groups according to the median metabolic pathway levels for comparative analysis. The correlation between the expression of drug resistance subgroup markers and the Lat_Resi gene subset was assessed. Tumor drug resistance subgroup marker genes were selected based on criteria of adj.p<0.05 and |log2FC|>0.25 for subsequent protein interaction network analysis, utilizing a high confidence threshold of 0.7.

### 2.6 Clinical samples and immunohistochemistry analysis

Paraffin-embedded tissues from patients diagnosed with ovarian serous cancer between 2018 and 2021 were collected. All patients received primary surgery, subsequently followed by carboplatin-based chemotherapy completion. **Table 2** presents the characteristics of the patients. The patients were categorized into two groups: the chemoresistant group and the sensitive group, defined as follows: Drug resistance was observed during postoperative chemotherapy, as the disease continued to progress. CA125 levels did not return to the normal range following six courses of chemotherapy, and relapse occurred within six months after the initial treatment. Chemotherapy sensitivity: recurrence or non-recurrence after a six-month period. Prior to the survey, participants were briefed on the study’s purpose and provided informed consent. The Ethics Committee of Shengjing Hospital approved this study.

**Table 2:** Patients’ characteristics.

	Resistant	Sensitive	Overall	P-value
	(N=32)	(N=40)	(N=72)	
Age				
Mean (SD)	55.4 (8.76)	53.1 (10.1)	54.1 (9.56)	0.299
Median [Min, Max]	56.0 [34.0, 76.0]	52.0 [24.0, 78.0]	52.0 [24.0, 78.0]	
FIGO stage				
I	2 (6.3%)	7 (17.5%)	9 (12.5%)	0.403
II	3 (9.4%)	6 (15.0%)	9 (12.5%)	
III	26 (81.3%)	26 (65.0%)	52 (72.2%)	
IV	1 (3.1%)	1 (2.5%)	2 (2.8%)	
Grade				
G1	1 (3.1%)	0 (0%)	1 (1.4%)	0.106
G2	16 (50.0%)	11 (27.5%)	27 (37.5%)	
G3	12 (37.5%)	20 (50.0%)	32 (44.4%)	
G4	3 (9.4%)	9 (22.5%)	12 (16.7%)	

IHC was performed in accordance with previous standard operating procedures (13, 14). IHC with antibodies against S100A4 (#16105-1-AP, 1:200, ThermoFisher) and ALDH1A1 (#ab195254, 1:100, Abcam) was performed to detect protein expression levels. Randomly selected images of each region were taken at 40x magnification. For evaluation of IHC findings, scoring of immunoreactivity was performed, on the basis of the percentage of immunopositive cells and the immunointensity, with multiplication of values of the two parameters, as described previously (14). Each section was assessed independently by two investigators who were unaware of the clinical details of the OC patients. To assess the prognostic significance of S100A4 and ALDH1A1 expression, scores were categorized into two groups (high and low) using the median values as the cutoff for each category.

### 2.7 Cell culture and establishment of drug-resistant cell lines

Platinum-sensitive human ovarian papillary serous adenocarcinoma cell line OV-90 (ATCC#CRL11732) was cultured in RPMI medium with 10% FBS and 1% penicillin-streptomycin according to manufacturers’ instructions. Cells were subjected to treatment with escalating concentrations of cisplatin (DDP, IC0440, Beijing Solarbio Science and Technology Co.), specifically 10, 20, 40, 80, 100, and 120μM to generate chemoresistant OV-90/DDP cell lines. Cisplatin at a concentration of 10 μM was introduced when the cells reached the exponential phase, with subsequent concentrations administered at the same phase until a final concentration of 120 μM was achieved. The culture conditions of the cell lines were preserved in accordance with those of the original OV-90 cell line during the entire culture period. Cells from each concentration phase were cultured with platinum drugs for a minimum of two weeks, with medium changes occurring every two days. Cisplatin-resistant subclones were generated by intermittently exposing cells to 2 μM cisplatin while maintaining a concentration of 100 μM to sustain chemoresistant clones. The concentration indicates the threshold that cells can endure without compromising reproductive viability. The resistant cell line OV-90/DDP was established after 12 months of induction. The cell line tested negative for mycoplasma contamination.

### 2.8 Cell survival assays

Cells (1 × 10^4^ cells/well) were plated in 96-well plates and incubated for 24 hours. The cells were subjected to varying concentrations of cisplatin (0-120 µM). Following a 72-hour incubation period, cell viability was assessed using the MTT assay in accordance with the manufacturer’s instructions (Sigma Aldrich). Inhibitory concentrations (ICs) were calculated from three independent experiments, and the IC50 values were analyzed using GraphPad Prism software (v10.0.3, GraphPad Software Inc., San Diego, CA, USA).

### 2.9 Immunofluorescence analysis

OV-90 and OV-90/DDP cells were cultured in cell culture dishes at a density of 1 × 10^4^ cells/cm², achieving 80% confluence. The cells underwent fixation with 4% paraformaldehyde for 15 minutes and were subsequently permeabilized using 0.3% Triton X-100 (Solarbio, China) at 25°C for 10 minutes. Following a 30-minute blocking with 1% BSA in PBS, the cells were co-stained with primary antibodies: anti-Pan lactylation polyclonal antibody (PanKla, #PTM-1401, 1:1000, PTM BIO), anti-S100A4 (#16105-1-AP, 1:1000, ThermoFisher), and anti-YY1 (#46395S, 1:1000, Cell Signaling) for 1 hour. Cells were stained with Alexa Fluor 488 AffiniPure (1:50; A8807110; Yeasen) to label S100A4 and YY1 antibodies, and with Alexa Fluor 555 AffiniPure goat (1:100; A9825370; Yeasen) for Pan-Kla antibody labeling, at 4 °C overnight in the dark following thorough washing with PBS. The nucleus was stained using 4′,6-diamidino-2-phenylindole (DAPI; Boster Biological Technology, Wuhan, China). Fluorescence signals were detected via fluorescence microscopy (Olympus; Tokyo, Japan).

### 2.10 Western blot analysis

Total protein was isolated from cells utilizing RIPA buffer (10×; Cell Signaling Technology) and quantified with a BCA assay kit (Beyotime, China). Proteins were separated using 10% SDS-PAGE and subsequently transferred to PVDF membranes (Thermo Fisher Scientific). Membranes were blocked using 5% skim milk and incubated overnight at 4 °C with primary antibodies targeting PanKla (#PTM-1401, 1:1000, PTM BIO), S100A4 (#16105-1-AP, 1:1000, ThermoFisher), and β-actin (#4967, 1:5000, Cell Signal). The membranes were subsequently washed three times and incubated with HRP-conjugated secondary antibody (Beyotime, China) for a duration of 2 hours. Blots were visualized using an enhanced chemiluminescence substrate kit.

### 2.11 Statistics analysis

Statistical analyses were performed utilizing R software (version 4.4.0) and RStudio (version 2024.04.1 + 748) on a Mac computer. Visualizations were generated utilizing ggplot2 version 3.4.3 and GraphPad Prism version 10.0.3. All cell experiments were conducted in triplicate. The data are expressed as means ± SD and analyzed using an unpaired two-tailed Student’s t-test. Overall survival (OS) was defined as the interval from onset to death or the date of the latest follow-up assessment, and was assessed using Kaplan-Meier methods, with statistical comparisons performed via the log-rank test. A P-value less than 0.05 was deemed indicative of a statistically significant difference.

## 3 Results

### 3.1 Bulk RNA-Sequencing Analysis of lactylation-related resistance genes in OC

**Figure 1:**
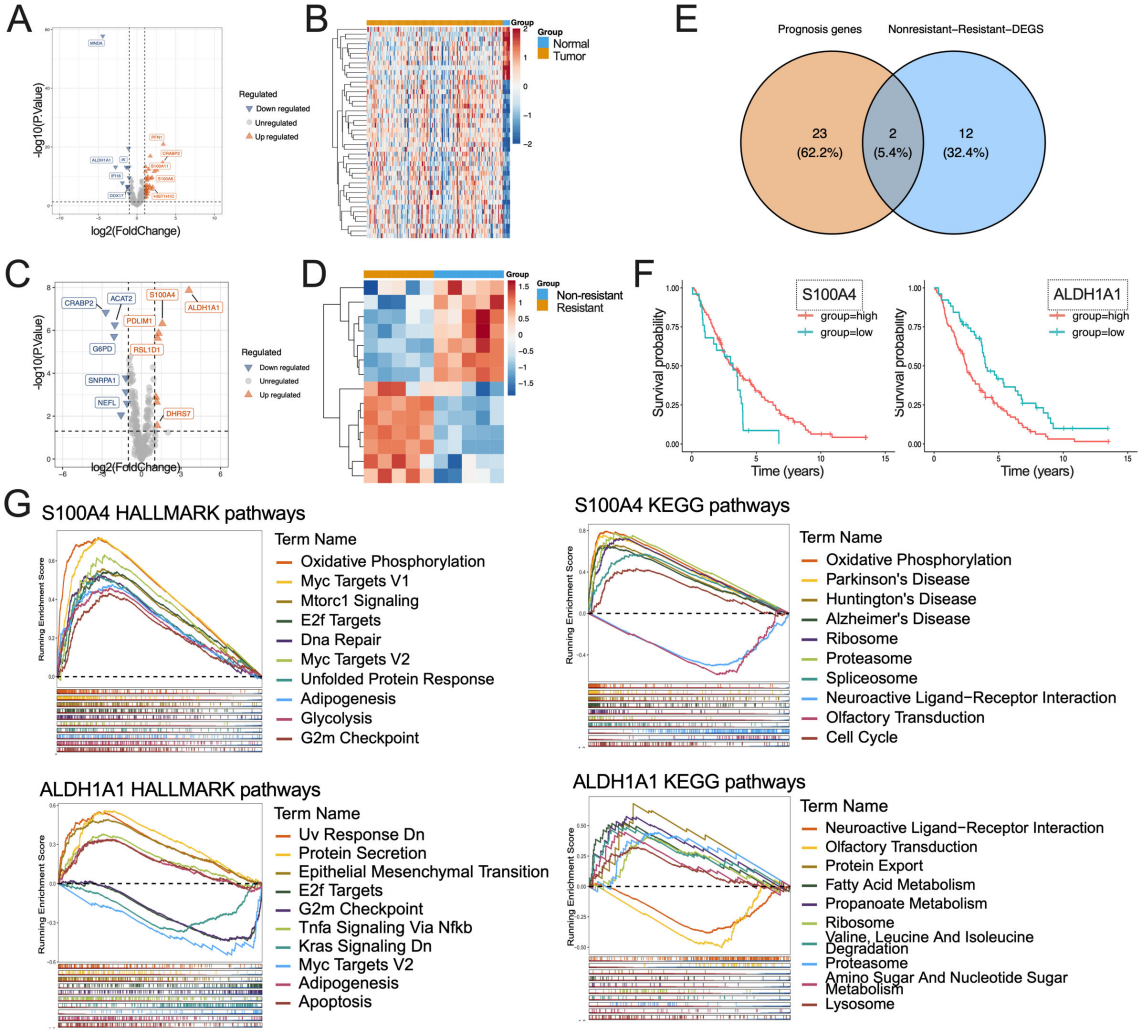
The exploration of potential lactylation-related chemotherapy-resistant genes in OC. **(A, B)** Volcano plot **(A)** and gene heatmap **(B)** of d DEGs for GSE26712 dataset; **(C, D)** Volcano plot **(C)** and gene heatmap **(D)** of DEGs for GSE15372 dataset; **(E)** The DEGs between the resistance and non-resistance group were intersected with the prognostic LacRGs; **(F)** Kaplan Meier survival plot of ALDH1A1 and S100A4 in GSE26712 dataset; **(G)** Enrichment HALLMARK and KEGG pathway analysis for S100A4 and ALDH1A1.

Initially, the disparities between the tumor and normal group were examined using the GSE26712 dataset in conjunction with the LacRGs. Applying the criteria of threshold, a total of 44 DEGs were identified, comprising 33 up- and 11 down-regulated genes (**Figures 1A, B**, **Supplementary Table 1**). Additionally, 25 prognostic LacRGs were identified through KM survival analysis (**Supplementary Table 2**).

Secondly, the analysis of differences between the resistance and the non-resistance group was conducted using the GSE15372 data in conjunction with the LacRGs. 14 DEGs were identified, comprising 7 up- and 7 down-regulated genes (**Figures 1C, D**, **Supplementary Table 3**). Two Lat_Resi genes were identified through the intersection with prognostic LacRGs (**Figure 1E**), specifically ALDH1A1 and S100A4 (**Figure 1F**, **Supplementary Table 4**).

**Figure 2:**
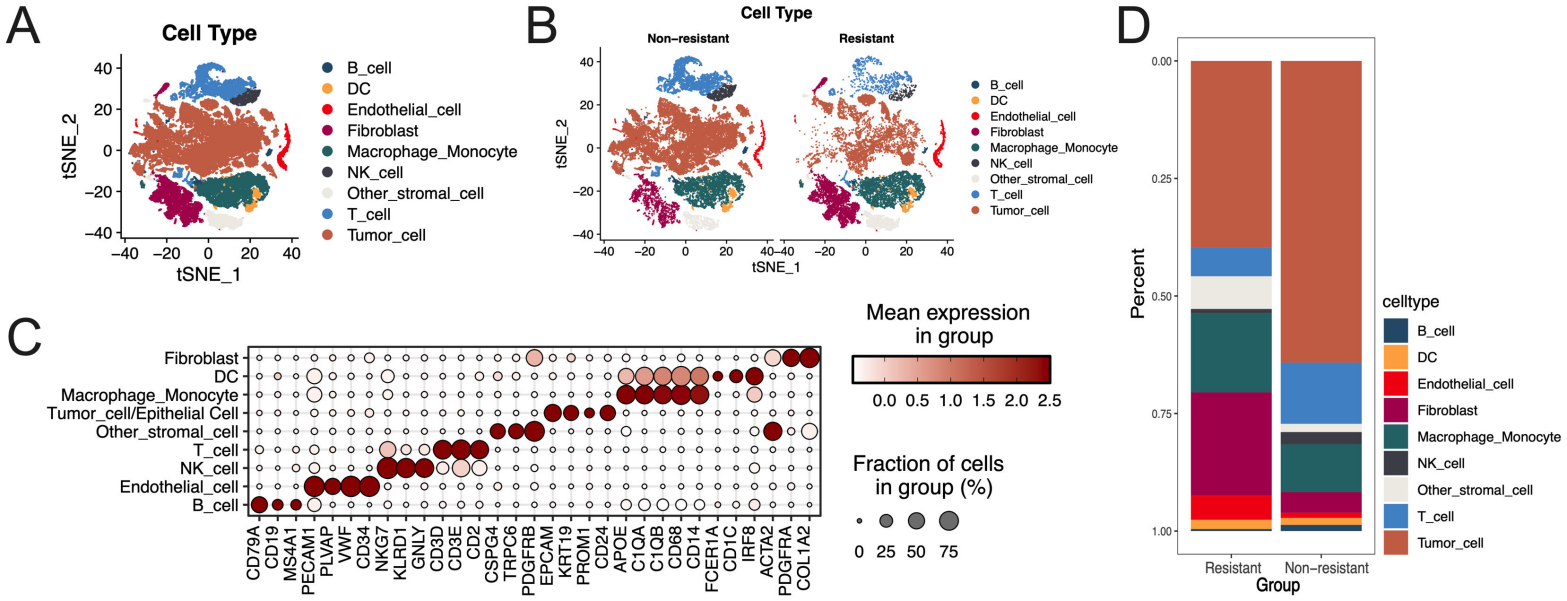
Cell annotation and different cell type distribution. **(A)** Cell type distribution for the scRNA sequence data; **(B)** Cell distribution between non-resistant and resistant groups; **(C)** Bubble plot of marker gene expression for each cell type; **(D)** Proportion of different cell types between resistant and non-resistant groups.

Single gene enrichment analysis was conducted using genes ALDH1A1 and S100A4 in conjunction with HALLMARK and KEGG gene sets. S100A4 enriched 31 and 46 items from the HALLMARK and KEGG databases, including Oxidative phosphorylation, glycolysis, and some important tumorigenesis pathways, such as: Myc Targets V1, Mtorc1 Signaling, DNA repair, cell cycle, etc. ALDH1A1 was associated with 27 HALLMARK items and 15 KEGG items, including metabolisms of fatty acids, propanoate, amino sugars, and nucleotide sugars, along with some tumorigenesis pathways such as Epithelial mesenchymal transition (EMT), E2f targets, Kras signaling, etc (**Figure 1G**, **Supplementary Table 5**).

### 3.2 scRNA-Sequencing Analysis of cell subgroups with chemotherapy response distribution

Initially, single cell data quality control was performed (**Supplementary Figure 1A**), and batch effects were mitigated using the Harmony package (**Supplementary Figure 1B**). Consequently, a total of 57,680 cells and 26,729 genes were included in the subsequent analysis. The top 30 principal component scores (PCS) were selected for further analysis (**Supplementary Figure 1C**), resulting in the identification of 21 clusters through clustering (**Supplementary Figure 1D**). Subsequently, cell type annotation, in conjunction with literature marker genes and the identification of tumor cells through additional clustering of epithelial cells and CNV (**Supplementary Figure 1E**), yielded nine distinct cell types, comprising 572 B cells, 1462 endothelial cells, and 572 B cells. In the study, the following cell counts were recorded: 7245 macrophage/monocyte, 1132 NK cells, 950 dendritic cells, 6186 fibroblasts, 31782 tumor cells, 2089 other stromal cells, and 6043 T cells. The t-SNE analysis illustrates the distribution of various cell types (**Figure 2A**), the differentiation of cells between resistant and non-resistant groups (**Figure 2B**), and the expression distribution of marker genes for each cell type (**Figure 2C**). We examined the proportions of cells in both the drug resistance group and the non-drug resistance group (**Figure 2D**). The analysis revealed a high proportion of tumor cells in both groups; however, the drug resistance group exhibited a higher proportion of fibroblasts, macrophages/monocytes, and endothelial cells.

### 3.3 Identification of resistance subgroup in tumor cells

**Figure 3:**
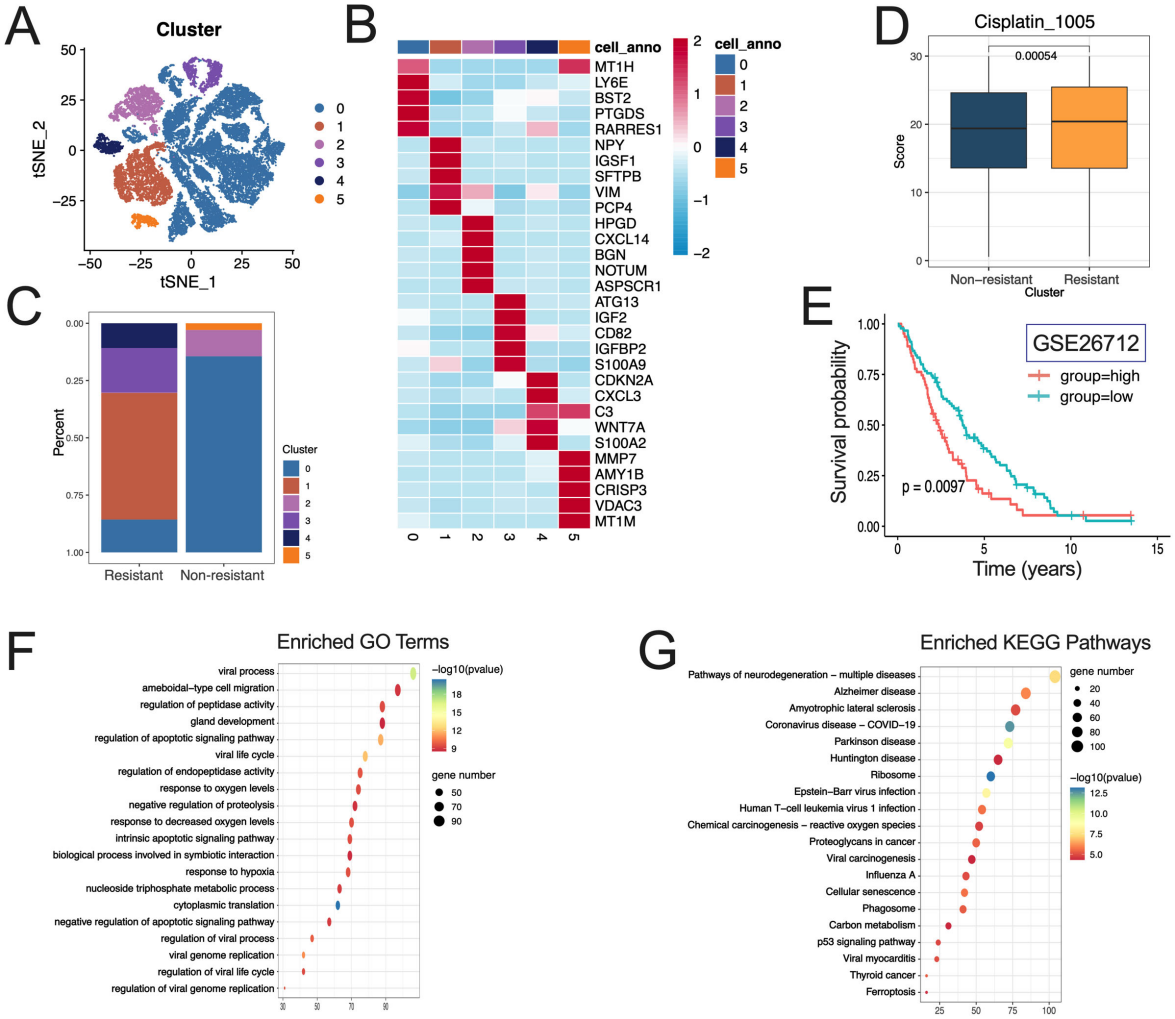
Identification of resistant subgroup in tumor cells and functional characteristics. **(A)** Distribution of tumor cell clusters; **(B)** Expression of selected marker genes in each subcluster; **(C)** Distribution of clusters between the resistant and non-resistant groups; **(D)** Distribution of IC50 values of cisplatin in resistant and non-resistant subgroup; **(E)** KM survival curves for the resistant subgroup; F.G. GO **(F)** and KEGG **(G)** enrichment analysis results for DGEs between resistance and non-resistance subgroup.

The tumor cells were classified into six distinct clusters (**Figure 3A**). The expression levels of selected marker genes in each sub-cluster are presented (**Figure 3B**, **Supplementary Table 6**), along with the cell proportions between the resistant and non-resistant groups (**Figure 3C**). Based on the distribution of various clusters across the two groups, clusters 0, 2, and 5 were identified as non-resistant subsets, while clusters 1, 3, and 4 were classified as resistant subsets. DEGs between the 2250 resistant subgroups and non-resistant subgroups were identified (**Supplementary Table 7**).

The IC50 values of chemotherapy drugs in each tumor cell were analyzed, revealing a significant difference in the IC50 value of cisplatin between the resistant and non-resistant subgroups (P<0.05). Specifically, the IC50 value in the resistant group was higher than that in the non-resistant subgroup (**Figure 3D**). The score of chemoresistant subgroups derived from GSE26712 data, in conjunction with marker genes of these subgroups, was utilized and categorized based on the optimal threshold value. The prognostic Kaplan-Meier survival curve indicates that resistant subgroups are associated with a poor prognosis in tumors (**Figure 3E**).

GO and KEGG enrichment analyses, in conjunction with marker genes from various clusters, revealed that 841, 1323, 1530, 1392, and 1623 entries were enriched for GO across tumor subgroups 0, 1, 2, 3, 4, and 5, respectively. The regulation of peptidase activity and the NADH dehydrogenase complex assembly are included. In KEGG, tumor subgroup 0 cluster, 1 cluster, 2 cluster, 3 cluster, 4 cluster, and 5 cluster were enriched with 58, 43, 82, 82, and 93 entries, respectively. Incorporating the NOD−like receptor signaling pathway, glycolysis/gluconeogenesis, and sphingolipid metabolism (**Supplementary Figure 2**, **Supplementary Table 8**).

In conjunction with the DEGs identified between the resistant and non-resistant subgroups, GO and KEGG enrichment analyses revealed 2165 enriched GO entries and 106 enriched KEGG entries, respectively, incorporating response to oxygen/hypoxia, carbon metabolism, proteoglycans in cancer, and the p53 signaling pathway (**Figures 3F, G**, **Supplementary Table 9**).

### 3.4 Clarification of the mechanism underlying lactylation-mediated resistance in resistant subsets

**Figure 4:**
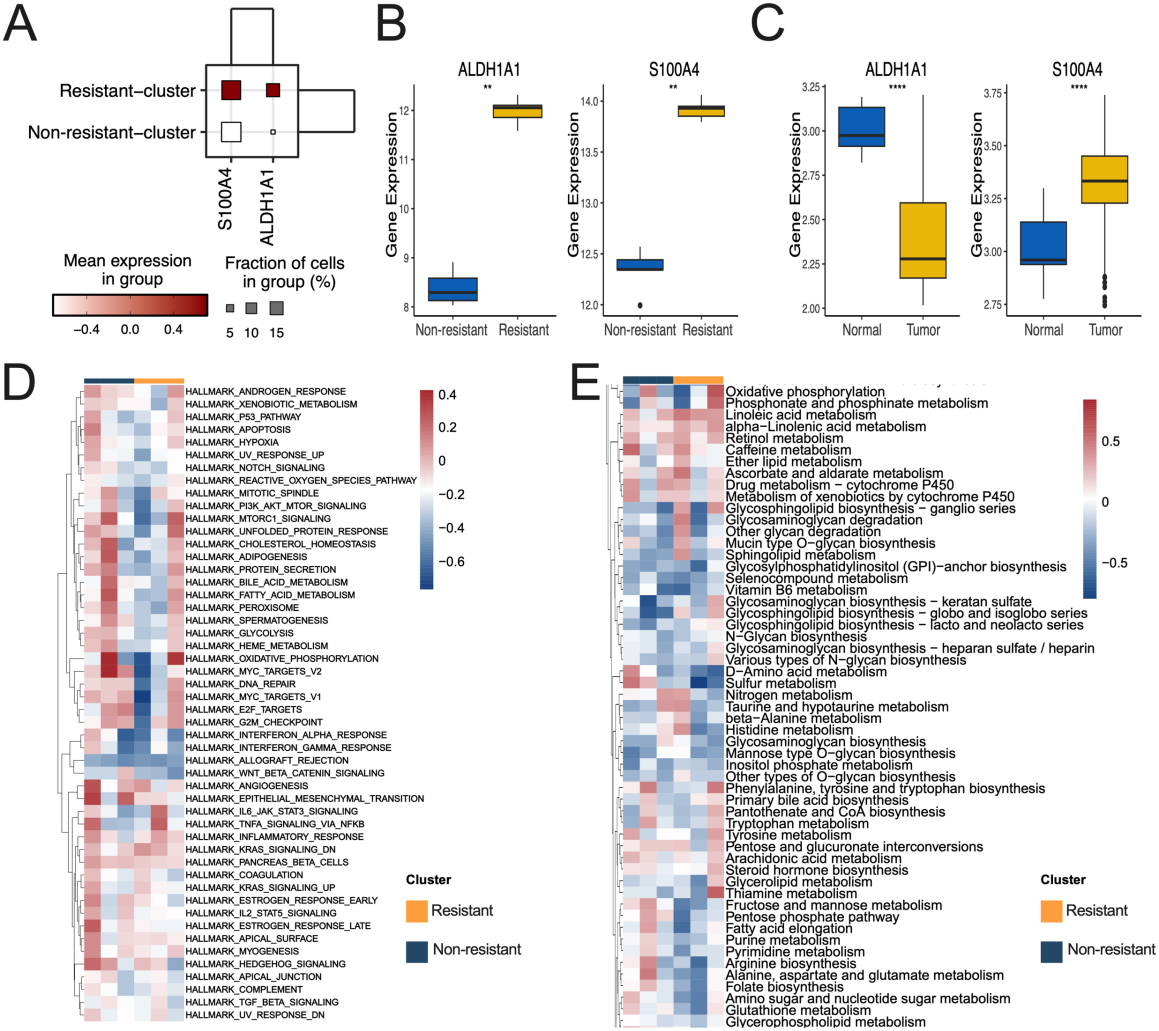
Distribution of expression for tumor and metabolism-related pathways in resistant and non-resistant subgroups. **(A)** ALDH1A1 and S100A4 expression distribution in resistant and non-resistant subgroups in scRNA sequence. **(B, C)** ALDH1A1 and S100A4 expression distribution in resistant and non-resistant subgroups **(B)**, as well as tumor and normal subgroups **(C)** in bulk RNA sequence. **(D, E)** Panels D and E present a heatmap illustrating the enriched Hallmark tumor pathway **(D)** and the score differences in the KEGG metabolic pathway **(E)** between resistant and non-resistant subsets. (**p<0.01, ****p<0.0001).

Analysis of the expression levels of ALDH1A1 and S100A4 in each tumor cell subset revealed that the chemoresistant tumor cell subset exhibited elevated levels (**Figure 4A**). The expression patterns of ALDH1A1 and S100A4 aligned with the overall transcriptome data, showing elevated levels in the chemoresistant group (**Figure 4B**). Specifically, ALDH1A1 exhibited reduced expression in the tumor group, whereas S100A4 levels were increased in the tumor group (**Figure 4C**).

Enrichment scores for metabolic and immune pathways in each tumor cell were calculated, revealing that the OXIDATIVE_PHOSPHORYLATION and TNFA_SIGNALING_VIA_NFKB pathways exhibited greater enrichment in the resistant subgroup (**Figure 4C**). Linoleic acid metabolism, Phosphonate and phosphinate metabolism, Oxidative phosphorylation and other metabolic pathways exhibited greater enrichment in the resistant subgroup (**Figure 4D**).

**Figure 5:**
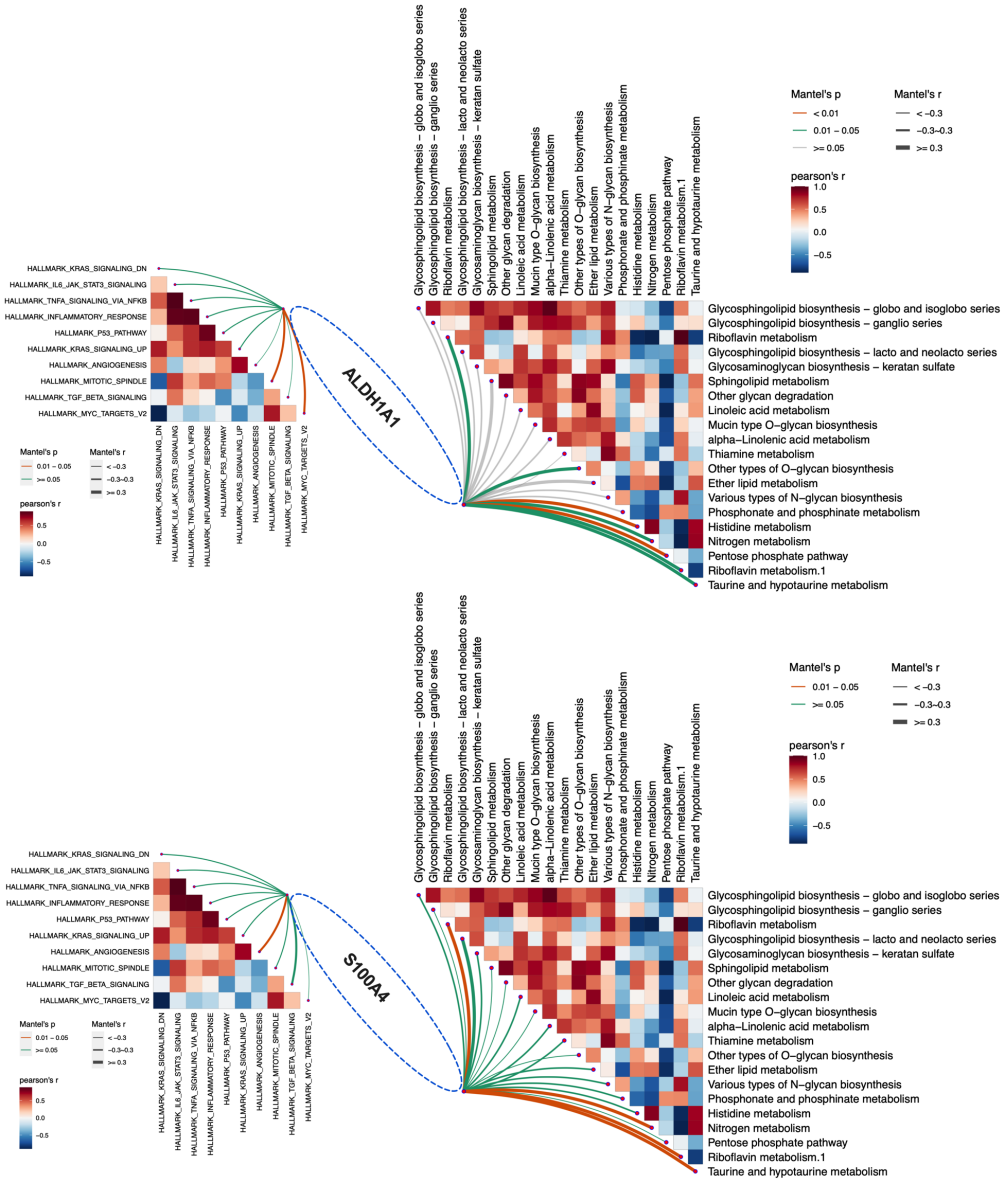
The relationship between ALDH1A1, S100A4 and enhanced immune and metabolic pathways in the resistant subgroup.

The correlation analysis of ALDH1A1 with the up-regulated immune and metabolic pathways in the resistant subset indicated a significant association with HALLMARK_MITOTIC_SPINDLE and HALLMARK_MYC_TARGETS_V2. Additionally, ALDH1A1 demonstrated a significant correlation with histidine metabolism and the pentose phosphate pathway (**Figure 5**). S100A4 exhibited a notable association with HALLMARK_ANGIOGENESIS and Mannose type O−glycan biosynthesis. Additionally, a significant correlation was identified between nitrogen metabolism and riboflavin metabolism, along with a relationship between taurine and hypotaurine metabolism (**Figure 5**).

### 3.5 Lactylation-related metabolic abnormalities mediate chemoresistance

**Figure 6:**
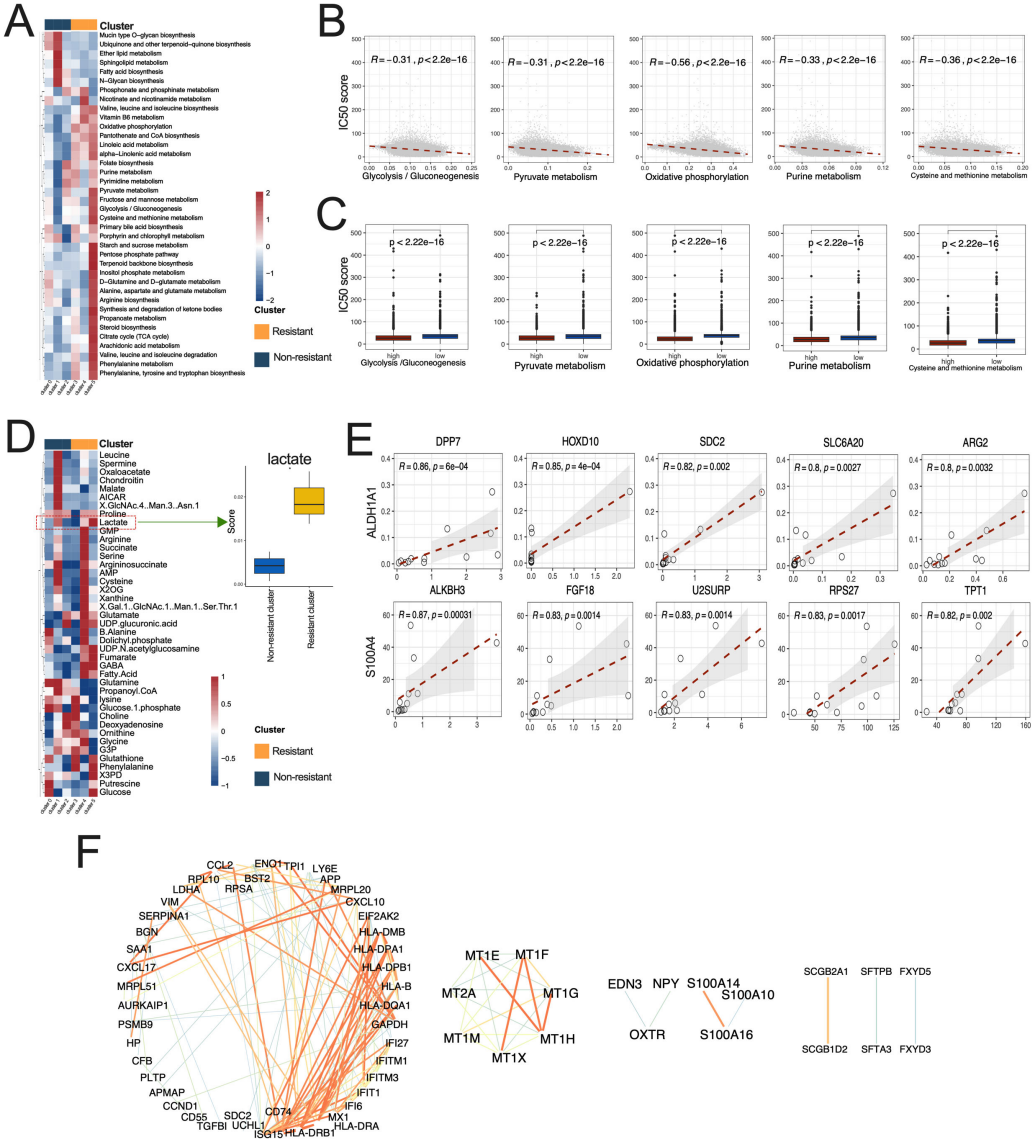
Metabolic abnormalities associated with lactylation in resistant and non-resistant subgroups. **(A)** The heatmap illustrated the variation in metabolic pathway scores between resistant and non-resistant tumor cell subgroup. **(B)** Correlation of various metabolic pathways with IC50 values; **(C)** Correlation between high and low levels of various metabolic pathways and IC50; **(D)** Left panel: Distribution of lactate products. Right panel: Heatmap illustrating the differences in metabolite scores between resistant and non-resistant of tumor cell subgroup. **(E)** Correlation between Top5 marker genes and the expression of ALDH1A1 and S100A4. **(F)** Protein-protein interaction network of marker genes associated with drug resistance subgroups.

A comparative analysis was conducted using ScMetabolism to quantify the scores of metabolic pathways in various tumor cell subgroups, specifically between resistant and non-resistant subgroups. Metabolic pathways including Glycolysis/Gluconeogenesis, the Citrate cycle (TCA cycle), Phenylalanine metabolism, and the Pentose phosphate pathway exhibited higher scores in resistant subgroups. Conversely, pathways such as fatty acid biosynthesis and sphingolipid metabolism demonstrated elevated scores in non-resistant subgroups (**Figure 6A**, **Supplementary Figure 3**).

Following the quantification of metabolic pathway levels in each tumor cell, the cells were categorized into groups based on high and low metabolic pathway levels. The correlation results (P<0.01, | Correlation |>0.3) demonstrate a negative correlation between the levels of various metabolic pathways (Glycolysis/Gluconeogenesis, Pyruvate Metabolism, Purine Metabolism, Oxidative Phosphorylation, Cysteine and Methyline Metabolism) and the IC50 of cisplatin (**Figures 6B, C**).

scFEA analysis revealed a higher enrichment of lactate in the resistant subgroup products (**Figure 6D**);

Correlation analysis was performed between the marker genes of chemoresistant subgroups and ALDH1A1 and S100A4 (**Supplementary Table 10**). The top five genes exhibiting significant positive correlations are presented in **Figure 6E**. Analysis of the protein-protein interaction network was conducted on the marker genes of resistant subgroups (**Figure 6F**, **Supplementary Table 11**), comprising 140 nodes and 144 edges.

### 3.6 Increased S100A4 and ALDH1A1 are associated with chemoresistance in OC

**Figure 7:**
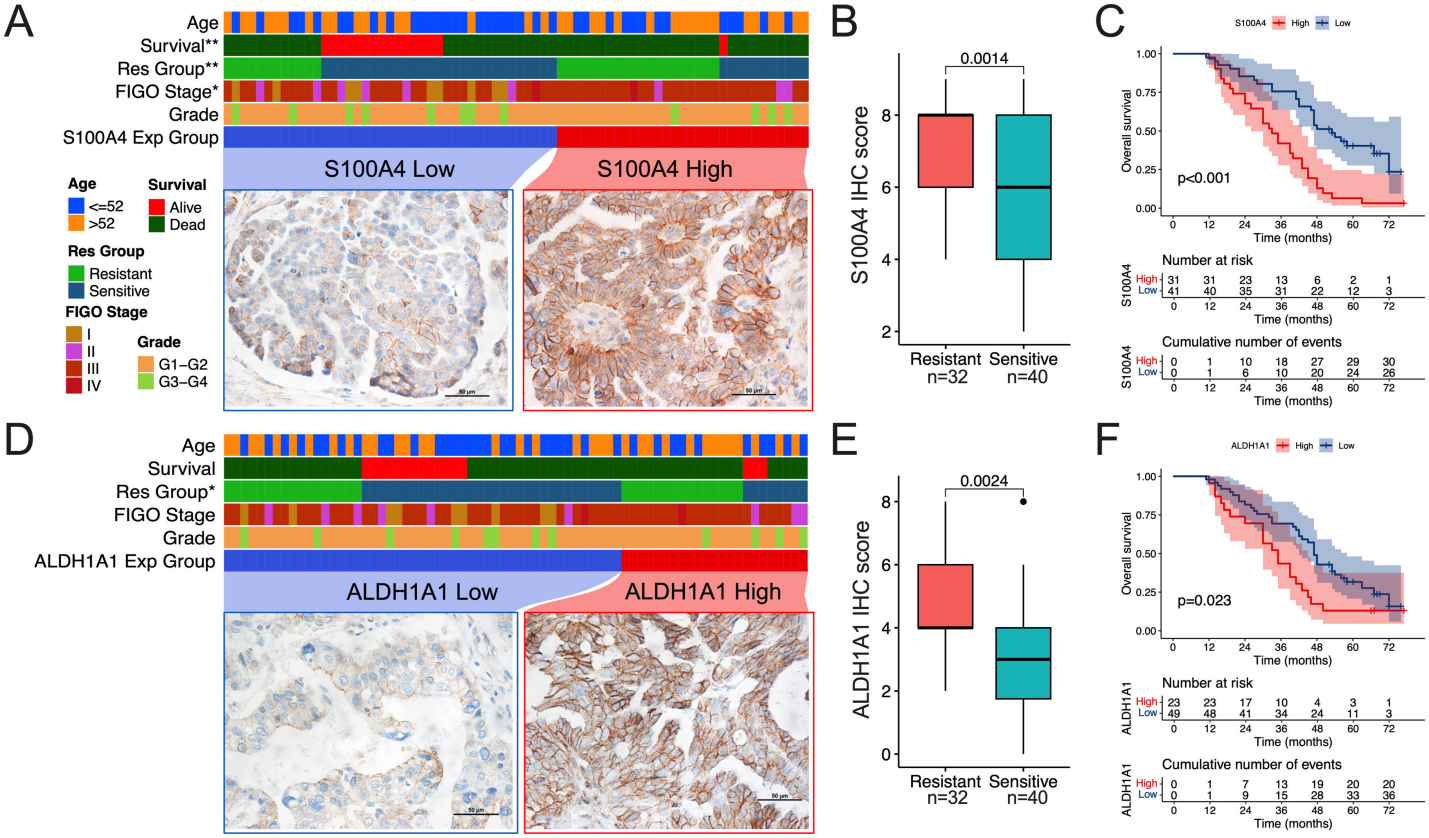
S100A4 and ALDH1A1 are elevated in chemoresistant OC tissues. **(A, D)** Heatmaps showing the relationship between low and high expression levels of S100A4 **(A)** and ALDH1A1 **(D)** with survival, research group, FIGO stage, and grade in patients with serous OC (n = 72). **(B, E)** The comparison of IHC staining score for S100A4 **(B)** and ALDH1A1 **(E)** between resistant and sensitive groups. **(C, F)** The Kaplan-Meier survival analysis of overall survival for S100A4 **(C)** and ALDH1A1 **(F)** was stratified by the median IHC staining score. (*p<0.05, **p<0.01).

Previous research indicates that abnormal levels of S100A4 and ALDH1A1 correlate with chemotherapy response and survival outcomes in OC patients (15, 16). We examined the expression levels of S100A4 and ALDH1A1 in chemoresistant and chemosensitive OC tissues using IHC (**Table 3**, **Figure 7**). The levels of S100A4 and ALDH1A1 were significantly elevated in tissues from resistant patients compared to sensitive OC tissues (**Figures 7A, B, D, E**). The S100A4 score was notably higher in advanced clinical stages, while similar associations were not found for the ALDH1A1 score (**Table 3**). Kaplan-Meier curves indicated that OC patients exhibiting elevated S100A4 and ALDH1A1 scores experienced reduced overall survival compared to those with lower scores, with the trend for S100A4 being more pronounced (**Figures 7C, F**).

**Table 3:** Association of S100A4 and ALDH1A1 expression with clinical features of ovarian serous carcinoma.

Factor	S100A4 expression			ALDH1A1 expression		
	High (score>6)	Low (score ≤ 6)		High (score>4)	Low (score ≤ 4)	
	n (%)	n (%)	P-value	n (%)	n (%)	P-value
Age						
≤52 years	13 (41.9%)	24 (58.5%)	0.247	10 (43.5%)	27 (55.1%)	0.505
>52 years	18 (58.1%)	17 (41.5%)		13 (56.5%)	22 (44.9%)	
Research group						
Resistant	20 (64.5%)	12 (29.3%)	0.00613	15 (65.2%)	17 (34.7%)	0.0296
Sensitive	11 (35.5%)	29 (70.7%)		8 (34.8%)	32 (65.3%)	
FIGO stage						
I	0 (0%)	9 (22.0%)	0.0312	0 (0%)	9 (18.4%)	0.169
II	3 (9.7%)	6 (14.6%)		3 (13.0%)	6 (12.2%)	
III	27 (87.1%)	25 (61.0%)		19 (82.6%)	33 (67.3%)	
IV	1 (3.2%)	1 (2.4%)		1 (4.3%)	1 (2.0%)	
Grade						
G1-G2	27 (87.1%)	33 (80.5%)	0.67	19 (82.6%)	41 (83.7%)	1
G3-G4	4 (12.9%)	8 (19.5%)		4 (17.4%)	8 (16.3%)	

### 3.7 High expression validation of S100A4 and lactylation in cisplatin resistance

**Figure 8:**
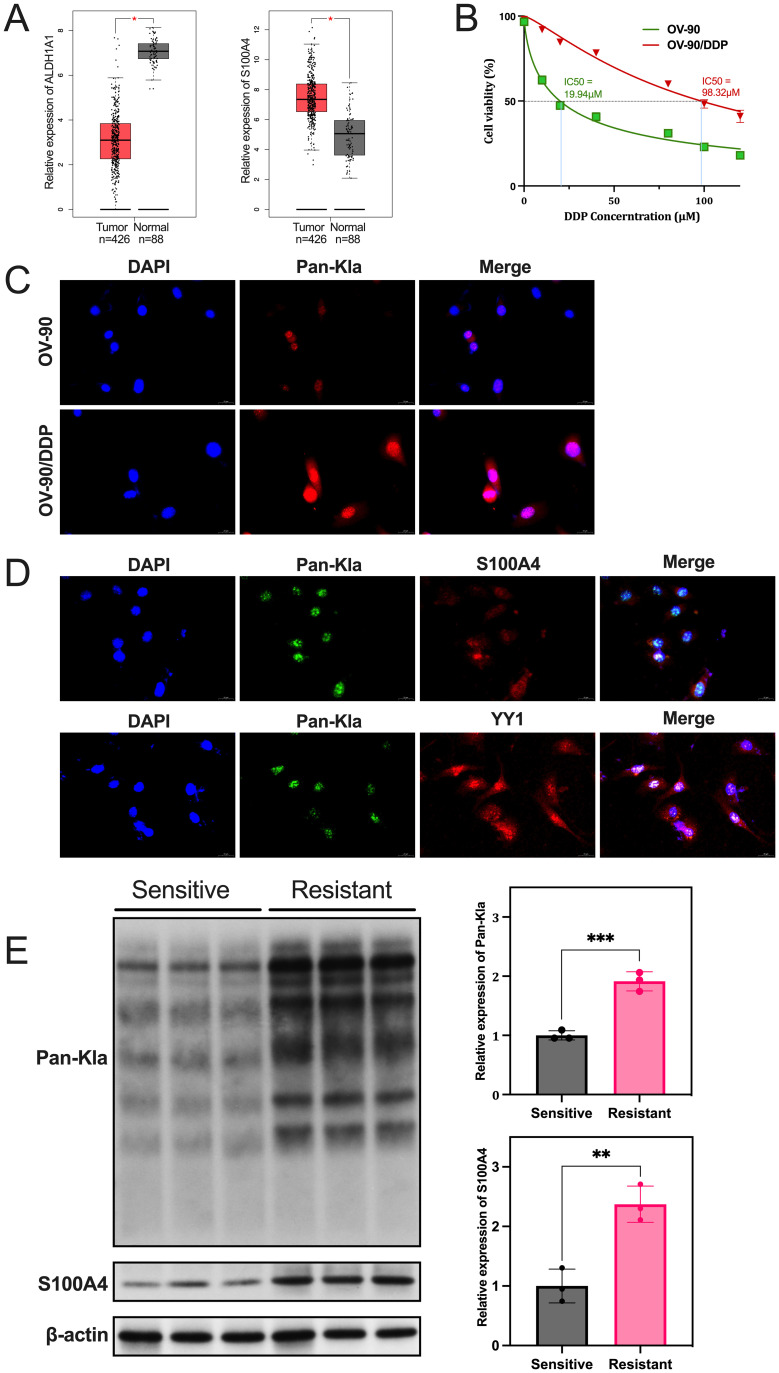
Expression levels of S100A4 and lactylation in OC cells. **(A)** Bioinformatics analysis of ALDH1A1 and S100A4 expression in OC, utilizing GEPIA, which includes 426 tumor samples and 88 normal ovarian tissues. **(B)** The inhibition ratio of each OC cell in the presence of DDP was assessed using the MTT assay. **(C)** Immunofluorescence staining was employed to assess the expression levels of Pan-Kla in sensitive and resistant OC cells. **(D)** Immunofluorescent staining of Pan-Kla (red), S100A4 (green), and their co-localization (yellow) in resistant cells, the positive control YY1 (green) also showed co-localization. **(E)** Western Blot analysis revealed significant differences in the expression of protein S100A4 and Pan-Kla between sensitive and resistant OC cells (*p<0.05, **p<0.01, ***p<0.001).

The Gepia online database revealed a significant difference in the expression of ALDH1A1 and S100A4 between ovarian normal tissues and cancer (**Figure 8A**). The increased expression of S100A4 in tumors necessitates further cytological experiments on this protein.

The cell viability across different DDP concentrations was evaluated, and the IC50 for drug resistance was established. OV-90/DDP cells demonstrated a DDP IC50 value of 98.32μM, surpassing the IC50 of sensitive cells OV-90, which was 19.94μM (**Figure 8B**). We analyzed the expression levels of Pan-Kla in two groups using the immunofluorescence method. The results indicated that resistant cells exhibited a more intense Pan-Kla signal compared to non-resistant cells (**Figure 8C**). Immunoconfocal microscopy demonstrated that S100A4 protein and Pan-Kla signals exhibited overlap in resistant OC OV-90/DDP cells, indicating co-localization of S100A4 and lactylation, the positive control of YY1 confirmed the aforementioned conclusion (**Figure 8D**). Western blot analysis was conducted to assess the expression levels of Pan-Kla and S100A4 protein in the two cell groups. Both the expression levels of Pan-Kla and S100A4 were significantly increased in resistant cells (all p<0.01, **Figure 8E**).

## 4 Discussion

The tumor microenvironment (TME) significantly influences the initiation and persistence of tumor drug resistance via mechanisms such as hypoxia, extracellular acidity, and the secretion of soluble factors like lactate (17, 18). The Warburg effect is a distinctive metabolic characteristic of cancer (19), characterized by the preference of cancer cells for anaerobic glucose metabolism over aerobic processes, resulting in significant lactate accumulation. Lactic acid, a byproduct of glycolysis, functions as a signaling molecule in several critical biological processes, including innate immunity (20), cell cycle regulation (21), energy support for tumor cells (22), and inhibition of immune cell cytotoxicity (23), etc. The elevated glycolytic metabolism observed in tumors correlates with resistance to treatment (18, 24). Research consistently investigates the role of lactate in tumor drug resistance. Prior studies indicate that lactate facilitates chemoresistance. In OC, MICU1 enhances lactate production, promotes tumor growth, induces cisplatin resistance, and decreases patient survival (25). In non-small cell lung cancer (NSCLC), lactate is identified as a critical factor in resistance to tyrosine kinase inhibitor (TKI) therapies (26). In colorectal cancer, B7-H3 increases glucose consumption and promotes hexokinase 2 (HK2) expression, leading to lactate production and enhanced resistance to platinum and 5-Fu (27).

In 2019, Professor Zhao Yingming’s team discovered that lactate induces lactylation on lysine residues as a post-translational modification (PTM) to regulate biological processes (28), functioning as an epigenetic modification that influences gene transcription (29). Lactylation of proteins represents a novel PTM that plays a significant role in the function of lactic acid. This discovery not only expands the scope of PTM research but also offers new avenues for investigating the role of lactate in areas such as oncology, inflammation, immunity, and targeted therapies (24). Currently, several writers and erasers of lactylation have been identified (28, 30). Although the precise mechanisms by which lactylation regulates biological activity remain unclear, it has been demonstrated to occur in various cell types and to play a crucial role in cellular processes (31). Recent studies have indicated that lactylation may promote tumor progression and contribute to drug resistance (32). The study revealed that OC cells from the resistant group exhibited elevated lactate levels, with lactate being enriched in the resistant subpopulation, consistent with findings in other tumor types.

Research on lactylation in OC remains limited, with few studies indicating that a lactylation-related gene model derived from the TCGA OC cohort can effectively predict prognosis and is associated with tumor classification and immunity (33). High expression of PanKla in the cytoplasm correlates with platinum-resistant recurrence, while protein lactylation may facilitate the migration of OC cells (34). Additionally, lactate promotes macrophage M2 polarization, enhances the proliferation and migration of OC cells, and contributes to tumorigenesis by activating CCL18 expression through H3K18 lactylation in macrophages (35). This study presents a systematic assessment of the mechanisms by which lactylation-related genes contribute to platinum resistance in OC, utilizing an integration of single-cell sequencing and bulk RNA-seq results for the first time. The findings indicate that lactate is more abundant in the resistant subpopulation products, suggesting that dysregulated lactylation-related metabolism may contribute to chemotherapy resistance in OC. The elevated lactylation levels of ALDH1A1 and S100A4 in tumor cell subgroups from the resistant group indicate that the lactylation of these genes is significant in the development of drug resistance. IHC and cytological experiments corroborated these findings, offering a significant reference for elucidating the lactylation-mediated drug resistance mechanism in OC.

Aldehyde dehydrogenase 1 family member A1 (ALDH1A1), as a key enzyme for retinoic acid biosynthesis and redox balance, is a biomarker of cancer stem-like cells (CSCs) (36). It can induce tumorigenesis by maintaining cancer stem cell properties, altering metabolism, and promoting DNA repair (37), and facilitating immune escape of tumor cells, for instance, through the ZBTB7B-glycolytic pathway (38). ALDH1A1 can also enhance the resistance of tumor cells to cisplatin and PARP inhibitors (39, 40). Currently, ALDH1A1-targeted therapy is extensively applied in cancer treatment (37, 41). In this study, we observed that ALDH1A1 was highly expressed in resistant tissues of OC and was correlated with the unfavorable prognosis of patients through tumorigenesis pathways such as EMT, Kras signaling, etc, which was similar to the previous research results (16). Moreover, single-cell data analysis indicated that the lactylation level was higher in the subgroups of resistant cells, suggesting that lactylation strengthened the ability of ALDH1A1 to promote drug resistance in OC. Further research is highly anticipated.

Multiple studies have shown the overexpression of S100A4 in tumor cells. Intracellular S100A4 facilitates proliferation, epithelial-mesenchymal transition (EMT), enhances the stemness of cancer cells, and promotes tumor metastasis (42, 43). It is also correlated with the adverse survival of tumor patients and serves as an excellent therapeutic target for tumors (44). S100A4 simultaneously promotes the differentiation of tumor-associated macrophages (TAMs) by enriching various cytokines, thereby shaping a TME that supports the survival of cancer cells (45). S100A4 regulates immunosuppressive T cells and myeloid cells in glioblastoma, the deletion of S100A4 in non-tumor cells is sufficient to reprogram the immune landscape and significantly enhance survival rates, indicating its potential as a target for immunotherapy (46). However, there are limited reports concerning its efficacy in drug resistance. Available reports suggest that S100A4 may enhance the resistance of pancreatic cancer cells to gemcitabine by suppressing apoptosis (47, 48), increase the resistance of colon cancer cells to MTX via the Wnt/β-catenin pathway (49), and its elevated expression correlates with docetaxel resistance in advanced prostate cancer (50), tamoxifen resistance in breast cancer (51), cisplatin resistance in laryngeal carcinoma (52), and drug resistance in leukemia (52). S100A4 may facilitate the enhanced recruitment of neutrophils, contributing to VEGF resistance in gliomas (53). In bulky stage (IB-IIA) cervical cancer, elevated S100A4 in stromal cells enhances chemotherapy sensitivity (54). The research indicates that S100A4 may play a role in regulating EMT or cancer stem cell properties in OC, thereby facilitating tumor progression via the miR-296/S100A4 (55) and S100A4/NMIIA/p53 (15) pathways. Limited research exists on the molecular mechanisms of S100A4 in OC chemoresistance. Only two studies have investigated this issue, indicating that elevated S100A4 expression in cancer cells may activate the NF-kB signaling pathway, inhibit p53 expression, and contribute to cisplatin resistance (56); Lung fibroblasts can stimulate the secretion of S100A4 in resistant OC cells via the IGF1R-α6 integrin-S100A4 pathway, resulting in its activation and enhancing the metastasis and colonization of late resistant cells (57). The findings of our study indicate that elevated levels of S100A4 in OC correlate with unfavorable prognosis and advanced stages, consistent with prior research (15, 55). S100A4 exhibits elevated expression in resistant tissues and cells, and it exists in a lactylation form, potentially enhancing its resistance properties. S100A4 may perform tumorigenesis roles through cancer pathways, such as glycolysis, Myc, Mtorc1 Signaling, DNA repair, etc. This offers a novel approach to investigating the platinum resistance mechanism of S100A4 in OC at the molecular level. Our study identified a correlation between ALDH1A1 and S100A4 with up-regulated immune and metabolic pathways in the resistant subgroup, alongside the enrichment of certain tumor and glucose metabolism-related pathways. Genes and interaction networks strongly correlated with ALDH1A1 and S100A4 were identified in the resistant subgroup. The findings offer substantial support for further investigation into the molecular mechanisms by which these two proteins facilitate platinum resistance via lactylation. The molecular mechanisms by which elevated lactate levels in the TME enhance ALDH1A1 or S100A4 lactylation to facilitate resistance in OC cells require further investigation. This includes identifying specific lactylation sites, discovering interacting proteins, and exploring underlying mechanisms. This research aims to enhance efforts to address OC resistance and improve the efficacy of targeted therapy.

In summary, this study systematically evaluated the mechanisms by which genes involved in lactylation promote platinum resistance in epithelial OC through the integration of single-cell sequencing and bulk RNA sequencing results. Further investigation into the mechanisms by which ALDH1A1 and S100A4 enhance platinum resistance in OC via lactylation is anticipated to provide significant insights into the resistance mechanisms of this disease.

## Data Availability

The raw data supporting the conclusions of this article will be made available by the authors, without undue reservation.

## Supplementary material

Supplementary Table 1 The differentially expressed LacRGs in OC.

Supplementary Table 2 The differentially expressed prognositic LacRGs in OC.

Supplementary Table 3 The differentially expressed resistance-related genes in OC.

Supplementary Table 4 The differentially prognostic gene related to resistance in OC.

Supplementary Table 5 The enriched HALLMARK and KEGG pathways in S100A4 and ALDH1A1.

Supplementary Table 6 Marker gene expression in different clusters.

Supplementary Table 7 Differential expressed genes between resistance subgroup and non-resistance subgroup.

Supplementary Table 8 The GO and KEGG enrichment analysis result for different clusters.

Supplementary Table 9 The GO and KEGG enrichment analysis result for resistance subgroup and non-resistance subgroup.

Supplementary Table 10 Results of correlation analysis between marker genes and ALDH1A1 and S100A4 expression in the resistant subgroup.

Supplementary Table 11 Protein-protein interaction network analysis result for marker genes in the resistant subgroup.

Supplementary Figure 1 Quality control for single cell data. **(A)** Quality control. **(B)** Reducing batch effects by Harmony package. **(C)** Top 30 principal component scores. **(D)** Different cluster. **(E)** Identifying tumor cells by cell type annotation.

Supplementary Figure 2 Single-cell data analysis showed functional enrichment analysis of marker genes in different clusters. **(A)** GO analysis. **(B)** KEGG analysis.

Supplementary Figure 3 Metabolism pathway score between resistance and non-resistance-subgroup.

## References

[1] Sideris M Menon U Manchanda R. Screening and prevention of ovarian cancer. Med J Aust. (2024) 220:264–74. doi: 10.5694/mja2.v220.5 PMC7617385 38353066

[2] St Laurent J Liu JF. Treatment approaches for platinum-resistant ovarian cancer. J Clin Oncol. (2024) 42:127–33. doi: 10.1200/JCO.23.01771 37910841

[3] Garcia-Canaveras JC Chen L Rabinowitz JD. The tumor metabolic microenvironment: lessons from lactate. Cancer Res. (2019) 79:3155–62. doi: 10.1158/0008-5472.CAN-18-3726 PMC6606343 31171526

[4] Hui S Ghergurovich JM Morscher RJ Jang C Teng X Lu W. Glucose feeds the TCA cycle via circulating lactate. Nature. (2017) 551:115–8. doi: 10.1038/nature24057 PMC5898814 29045397

[5] Tondo-Steele K McLean K. The “Sweet spot” of targeting tumor metabolism in ovarian cancers. Cancers (Basel). (2022) 14(19):4696. doi: 10.3390/cancers14194696 36230617 PMC9562887

[6] Zheng X Zhou Y Chen W Chen L Lu J He F. Ginsenoside 20(S)-rg3 prevents PKM2-targeting miR-324-5p from H19 sponging to antagonize the Warburg effect in ovarian cancer cells. Cell Physiol Biochem. (2018) 51:1340–53. doi: 10.1159/000495552 30481782

[7] Zhou Y Zheng X Lu J Chen W Li X Zhao L. Ginsenoside 20(S)-rg3 inhibits the Warburg effect via modulating DNMT3A/MiR-532-3p/HK2 pathway in ovarian cancer cells. Cell Physiol Biochem. (2018) 45:2548–59. doi: 10.1159/000488273 29558748

[8] Vathipadiekal V Wang V Wei W Waldron L Drapkin R Gillette M. Creation of a human secretome: A novel composite library of human secreted proteins: validation using ovarian cancer gene expression data and a virtual secretome array. Clin Cancer Res. (2015) 21:4960–9. doi: 10.1158/1078-0432.CCR-14-3173 25944803

[9] Li M Balch C Montgomery JS Jeong M Chung JH Yan P. Integrated analysis of DNA methylation and gene expression reveals specific signaling pathways associated with platinum resistance in ovarian cancer. BMC Med Genomics. (2009) 2:34. doi: 10.1186/1755-8794-2-34 19505326 PMC2712480

[10] Zheng X Wang X Cheng X Liu Z Yin Y Li X. Single-cell analyses implicate ascites in remodeling the ecosystems of primary and metastatic tumors in ovarian cancer. Nat Cancer. (2023) 4(8):1138–56. doi: 10.1038/s43018-023-00599-8 PMC10447252 37488416

[11] Cheng Z Huang H Li M Liang X Tan Y Chen Y. Lactylation-related gene signature effectively predicts prognosis and treatment responsiveness in hepatocellular carcinoma. Pharm (Basel). (2023) 16(5):644. doi: 10.3390/ph16050644 PMC10221268 37242427

[12] Ippolito L Morandi A Giannoni E Chiarugi P. Lactate: A metabolic driver in the tumour landscape. Trends Biochem Sci. (2019) 44:153–66. doi: 10.1016/j.tibs.2018.10.011 30473428

[13] Zhu LC Gao J Hu ZH Schwab CL Zhuang HY Tan MZ. Membranous expressions of Lewis y and CAM-DR-related markers are independent factors of chemotherapy resistance and poor prognosis in epithelial ovarian cancer. Am J Cancer Res. (2015) 5:830–43. PMC4396026 25973320

[14] Xu H Li X Wang S Li F Gao J Yan L. Multiomics analysis identifies key genes and pathways related to N6-methyladenosine RNA modification in ovarian cancer. Epigenomics. (2021) 13:1359–83. doi: 10.2217/epi-2021-0204 34550011

[15] Hiruta A Oguri Y Yokoi A Matsumoto T Oda Y Tomohiro M. S100A4/nonmuscle myosin IIA/p53 axis contributes to aggressive features in ovarian high-grade serous carcinoma. Am J Pathol. (2020) 190:2304–16. doi: 10.1016/j.ajpath.2020.07.014 32805233

[16] Izycka N Rucinski M Andrzejewska M Szubert S Nowak-Markwitz E Sterzynska K. The prognostic value of cancer stem cell markers (CSCs) expression-ALDH1A1, CD133, CD44-for survival and long-term follow-up of ovarian cancer patients. Int J Mol Sci. (2023) 24(3):2400. doi: 10.3390/ijms24032400 36768723 PMC9916537

[17] Qu Y Dou B Tan H Feng Y Wang N Wang D. Tumor microenvironment-driven non-cell-autonomous resistance to antineoplastic treatment. Mol Cancer. (2019) 18:69. doi: 10.1186/s12943-019-0992-4 30927928 PMC6441162

[18] de-la-Cruz-Lopez KG Castro-Munoz LJ Reyes-Hernandez DO Garcia-Carranca A Manzo-Merino J. Lactate in the regulation of tumor microenvironment and therapeutic approaches. Front Oncol. (2019) 9:1143. doi: 10.3389/fonc.2019.01143 31737570 PMC6839026

[19] Liao M Yao D Wu L Luo C Wang Z Zhang J. Targeting the Warburg effect: A revisited perspective from molecular mechanisms to traditional and innovative therapeutic strategies in cancer. Acta Pharm Sin B. (2024) 14:953–1008. doi: 10.1016/j.apsb.2023.12.003 38487001 PMC10935242

[20] Zhang W Wang G Xu ZG Tu H Hu F Dai J. Lactate is a natural suppressor of RLR signaling by targeting MAVS. Cell. (2019) 178:176–89.e15. doi: 10.1016/j.cell.2019.05.003 31155231 PMC6625351

[21] Liu W Wang Y Bozi LHM Fischer PD Jedrychowski MP Xiao H. Lactate regulates cell cycle by remodelling the anaphase promoting complex. Nature. (2023) 616:790–7. doi: 10.1038/s41586-023-05939-3 PMC12175651 36921622

[22] Icard P Shulman S Farhat D Steyaert JM Alifano M Lincet H. How the Warburg effect supports aggressiveness and drug resistance of cancer cells? Drug Resist Update. (2018) 38:1–11. doi: 10.1016/j.drup.2018.03.001 29857814

[23] Harmon C Robinson MW Hand F Almuaili D Mentor K Houlihan DD. Lactate-mediated acidification of tumor microenvironment induces apoptosis of liver-resident NK cells in colorectal liver metastasis. Cancer Immunol Res. (2019) 7(2):335–46. doi: 10.1158/2326-6066.CIR-18-0481 30563827

[24] Chen AN Luo Y Yang YH Fu JT Geng XM Shi JP. Lactylation, a novel metabolic reprogramming code: current status and prospects. Front Immunol. (2021) 12:688910. doi: 10.3389/fimmu.2021.688910 34177945 PMC8222712

[25] Chakraborty PK Mustafi SB Xiong X Dwivedi SKD Nesin V Saha S. MICU1 drives glycolysis and chemoresistance in ovarian cancer. Nat Commun. (2017) 8:14634. doi: 10.1038/ncomms14634 28530221 PMC5477507

[26] Apicella M Giannoni E Fiore S Ferrari KJ Fernandez-Perez D Isella. Increased lactate secretion by cancer cells sustains non-cell-autonomous adaptive resistance to MET and EGFR targeted therapies. Cell Metab. (2018) 28:848–65.e6. doi: 10.1016/j.cmet.2018.08.006 30174307

[27] Shi T Ma Y Cao L Zhan S Xu Y Fu F. B7-H3 promotes aerobic glycolysis and chemoresistance in colorectal cancer cells by regulating HK2. Cell Death Dis. (2019) 10(4):308. doi: 10.1038/s41419-019-1549-6 30952834 PMC6450969

[28] Zhang D Tang Z Huang H Zhou G Cui C Weng Y. Metabolic regulation of gene expression by histone lactylation. Nature. (2019) 574(7779):575–80. doi: 10.1038/s41586-019-1678-1 PMC6818755 31645732

[29] Sun L Zhang H Gao P. Metabolic reprogramming and epigenetic modifications on the path to cancer. Protein Cell. (2022) 13(12):877–919. doi: 10.1007/s13238-021-00846-7 34050894 PMC9243210

[30] Moreno-Yruela C Zhang D Wei W Baek M Liu W Gao J. Class I histone deacetylases (HDAC1-3) are histone lysine delactylases. Sci Adv. (2022) 8(3):eabi6696. doi: 10.1126/sciadv.abi6696 35044827 PMC8769552

[31] Liu Z Huang Y Liu X. Lactylation regulated DNA damage repair and cancer cell chemosensitivity. Sci Bull (Beijing). (2024) 69(9):1185–7. doi: 10.1016/j.scib.2024.02.037 38472020

[32] Li J Chen ZS Pan Y Zeng L. The important role of lactylation in regulating DNA damage repair and tumor chemotherapy resistance. Drug Resist Update. (2024) p:101148. doi: 10.1016/j.drup.2024.101148 39271382

[33] Yu L Jing C Zhuang S Ji L Jiang L. A novel lactylation-related gene signature for effectively distinguishing and predicting the prognosis of ovarian cancer. Transl Cancer Res. (2024) 13(5):2497–508. doi: 10.21037/tcr-24-319 PMC11170528 38881917

[34] Chao J Chen GD Huang ST Gu H Liu YY Luo Y. High histone H3K18 lactylation level is correlated with poor prognosis in epithelial ovarian cancer. Neoplasma. (2024) 71(4):319–32. doi: 10.4149/neo_2024_240127N41 39267539

[35] Sun J Feng Q He Y Wang M Wu Y. Lactate activates CCL18 expression via H3K18 lactylation in macrophages to promote tumorigenesis of ovarian cancer. Acta Biochim Biophys Sin (Shanghai). (2024) 56(9):1373–86. doi: 10.3724/abbs.2024111 PMC11543520 39010846

[36] Wang W He S Zhang R Peng J Guo D Zhang J. ALDH1A1 maintains the cancer stem-like cells properties of esophageal squamous cell carcinoma by activating the AKT signal pathway and interacting with beta-catenin. BioMed Pharmacother. (2020) 125:109940. doi: 10.1016/j.biopha.2020.109940 32044720

[37] Yue H Hu Z Hu R Guo Z Zheng Y Wang Y. ALDH1A1 in cancers: bidirectional function, drug resistance, and regulatory mechanism. Front Oncol. (2022) 12:918778. doi: 10.3389/fonc.2022.918778 35814382 PMC9256994

[38] Wang M Wang T Wang J Yang Y Li X Chen H. ALDH1A1 promotes immune escape of tumor cells through ZBTB7B-glycolysis pathway. Cell Death Dis. (2024) 15:568. doi: 10.1038/s41419-024-06943-9 39107297 PMC11303523

[39] Lavudi K Banerjee A Li N Yang Y Cai S Bai X. ALDH1A1 promotes PARP inhibitor resistance by enhancing retinoic acid receptor-mediated DNA polymerase theta expression. NPJ Precis Oncol. (2023) 7(1):66. doi: 10.1038/s41698-023-00411-x 37429899 PMC10333219

[40] Uddin MH Kim B Cho U Azmi AS Song YS. Association of ALDH1A1-NEK-2 axis in cisplatin resistance in ovarian cancer cells. Heliyon. (2020) 6(11):e05442. doi: 10.1016/j.heliyon.2020.e05442 33241139 PMC7672295

[41] Muralikrishnan V Fang F Given TC Podicheti R Chtcherbinine M Metcalfe TX. A novel ALDH1A1 inhibitor blocks platinum-induced senescence and stemness in ovarian cancer. Cancers (Basel). (2022) 14(14):3437. doi: 10.3390/cancers14143437 35884498 PMC9318275

[42] Kim B Jung S Kim H Kwon JO Song MK Kim MK. The role of S100A4 for bone metastasis in prostate cancer cells. BMC Cancer. (2021) 21(1):137. doi: 10.1186/s12885-021-07850-4 33549040 PMC7868026

[43] Chow KH Park HJ George J Yamamoto K Gallup AD Graber JH. S100A4 is a biomarker and regulator of glioma stem cells that is critical for mesenchymal transition in glioblastoma. Cancer Res. (2017) 77(19):5360–73. doi: 10.1158/0008-5472.CAN-17-1294 PMC5626628 28807938

[44] Fei F Qu J Zhang M Li Y Zhang S. S100A4 in cancer progression and metastasis: A systematic review. Oncotarget. (2017) 8(42):73219–39. doi: 10.18632/oncotarget.18016 PMC5641208 29069865

[45] Prasmickaite L Tenstad EM Pettersen S Jabeen S Egeland EV Nord S. Basal-like breast cancer engages tumor-supportive macrophages via secreted factors induced by extracellular S100A4. Mol Oncol. (2018) 12(9):1540–58. doi: 10.1002/mol2.2018.12.issue-9 PMC6120223 29741811

[46] Abdelfattah N Kumar P Wang C Leu JS Flynn WF Gao R. Single-cell analysis of human glioma and immune cells identifies S100A4 as an immunotherapy target. Nat Commun. (2022) 13(1):767. doi: 10.1038/s41467-022-28372-y 35140215 PMC8828877

[47] Mahon PC Baril P Bhakta V Chelala C Caulee K Harada T. S100A4 contributes to the suppression of BNIP3 expression, chemoresistance, and inhibition of apoptosis in pancreatic cancer. Cancer Res. (2007) 67(14):6786–95. doi: 10.1158/0008-5472.CAN-07-0440 17638890

[48] Ma G Sun Y Fu S. Evaluation of S100A4 mRNA in EUS-FNA specimens for the assessment of chemosensitivity to gemcitabine from patients with unresectable pancreatic cancer. Int J Clin Exp Pathol. (2015) 8(10):13284–8. PMC4680476 26722531

[49] Mencia N Selga E Rico I de Almagro MC Villalobos X Ramirez S. Overexpression of S100A4 in human cancer cell lines resistant to methotrexate. BMC Cancer. (2010) 10:250. doi: 10.1186/1471-2407-10-250 20515499 PMC2903526

[50] Zhu S Min Z Qiao X Chen S Yang J Zhang X. Expression profile-based screening for critical genes reveals S100A4, ACKR3 and CDH1 in docetaxel-resistant prostate cancer cells. Aging (Albany NY). (2019) 11:12754–72. doi: 10.18632/aging.102600 PMC6949054 31895690

[51] Bauerschmitz G Huchel S Gallwas J Grundker C. Inhibition of increased invasiveness of breast cancer cells with acquired tamoxifen resistance by suppression of CYR61. Cancer Genomics Proteomics. (2023) 20(6):531–8. doi: 10.21873/cgp.20403 PMC10614060 37889058

[52] Liang DP Huang TQ Li SJ Chen ZJ. Knockdown of S100A4 chemosensitizes human laryngeal carcinoma cells *in vitro* through inhibition of Slug. Eur Rev Med Pharmacol Sci. (2014) 18(22):3484–90. 25491625

[53] Liang J Piao Y Holmes L Fuller GN Henry V Tiao N. Neutrophils promote the Malignant glioma phenotype through S100A4. Clin Cancer Res. (2014) 20(1):187–98. doi: 10.1158/1078-0432.CCR-13-1279 PMC4422653 24240114

[54] Jin L Shen Q Ding S Jiang W Jiang L Zhu X. Immunohistochemical expression of Annexin A2 and S100A proteins in patients with bulky stage IB-IIA cervical cancer treated with neoadjuvant chemotherapy. Gynecol Oncol. (2012) 126(1):140–6. doi: 10.1016/j.ygyno.2012.04.005 22487537

[55] Yan W Chen J Chen Z Chen H. Deregulated miR-296/S100A4 axis promotes tumor invasion by inducing epithelial-mesenchymal transition in human ovarian cancer. Am J Cancer Res. (2016) 6:260–9. PMC4859658 27186401

[56] Li X Hou Y Han G Yang Y Wang S Lv X. S100A4/NF-kappaB axis mediates the anticancer effect of epigallocatechin-3-gallate in platinum-resistant ovarian cancer. iScience. (2024) 27(2):108885. doi: 10.1016/j.isci.2024.108885 38313051 PMC10835441

[57] Deo AN Thorat R Dhadve AC De A Rekhi B Ray P. IGF1R-alpha6 integrin-S100A4 network governs the organ-specific metastasis of chemoresistant epithelial ovarian cancer cells. Biochim Biophys Acta Mol Basis Dis. (2022) 1868(1):166282. doi: 10.1016/j.bbadis.2021.166282 34600083

