# Supplementary figures and images for: Integration of scRNA-seq and bulk RNA-seq to reveal the association and potential molecular mechanisms of metabolic reprogramming regulated by lactylation and chemotherapy resistance in ovarian cancer
Source: Front Immunol. 2025 Feb 28;16:1513806. doi: 10.3389/fimmu.2025.1513806 (PMC11907005; doi:10.3389/fimmu.2025.1513806)

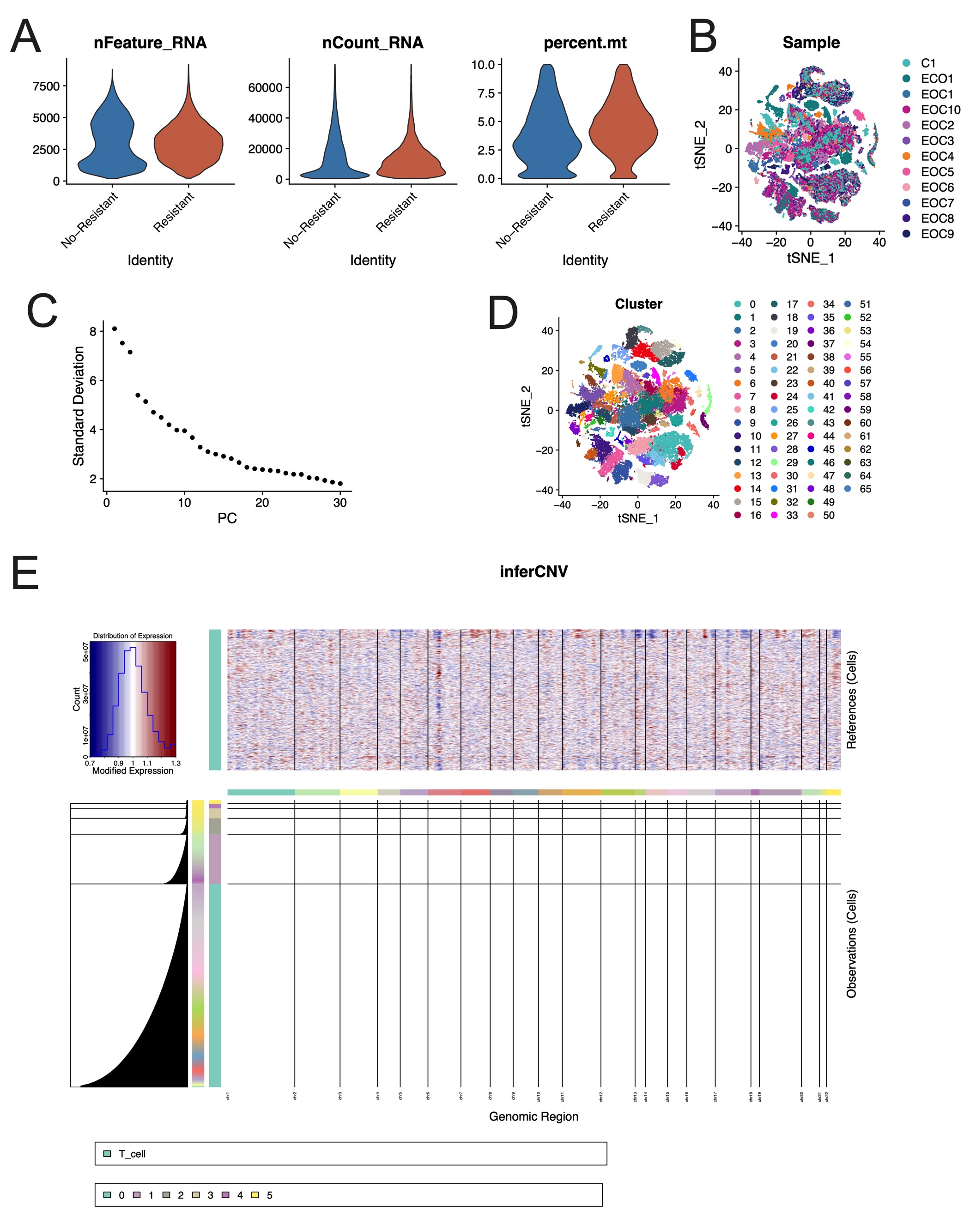

Supplement: Supplementary Figure 1 — Quality control for single cell data. (A) Quality control. (B) Reducing batch effects by Harmony package. (C) Top 30 principal component scores. (D) Different cluster. (E) Identifying tumor cells by cell type annotation. [file Image1.jpeg]

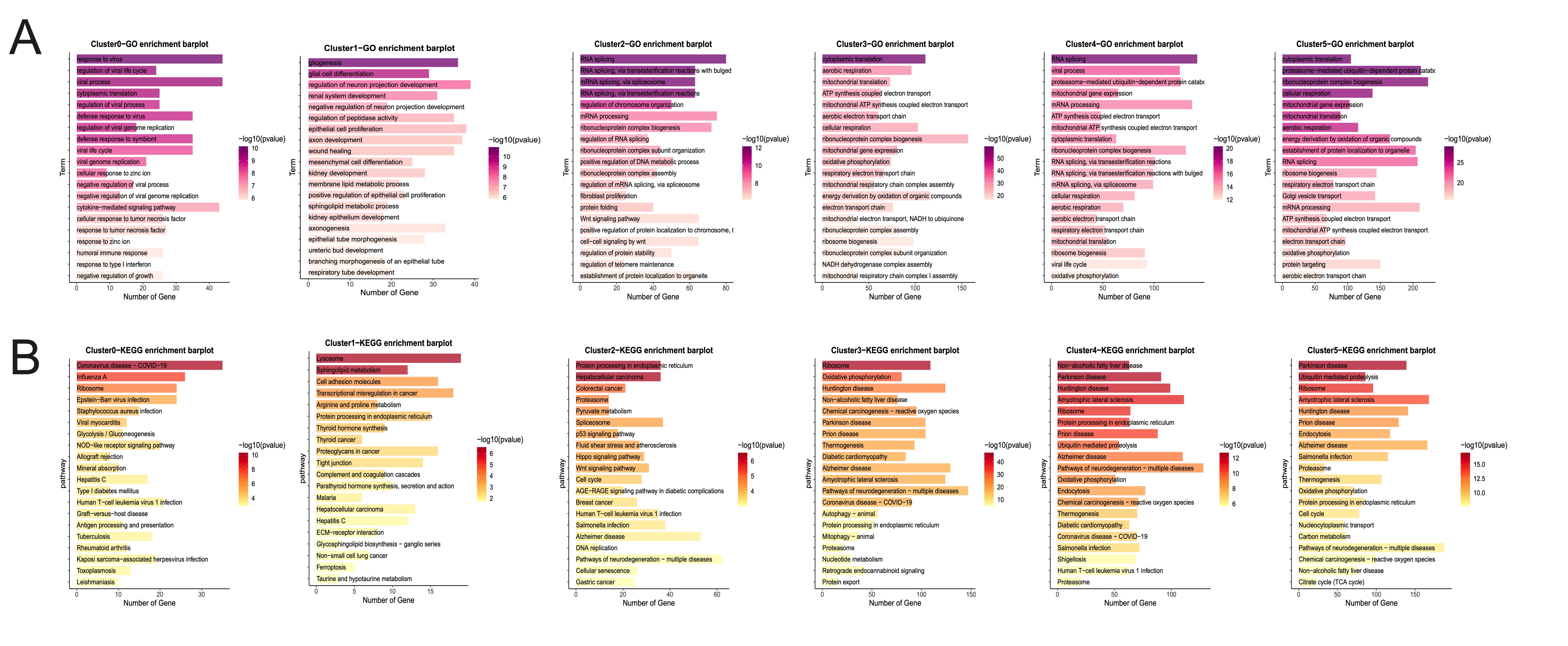

Supplement: Supplementary Figure 2 — Single-cell data analysis showed functional enrichment analysis of marker genes in different clusters. (A) GO analysis. (B) KEGG analysis. [file Image2.jpeg]

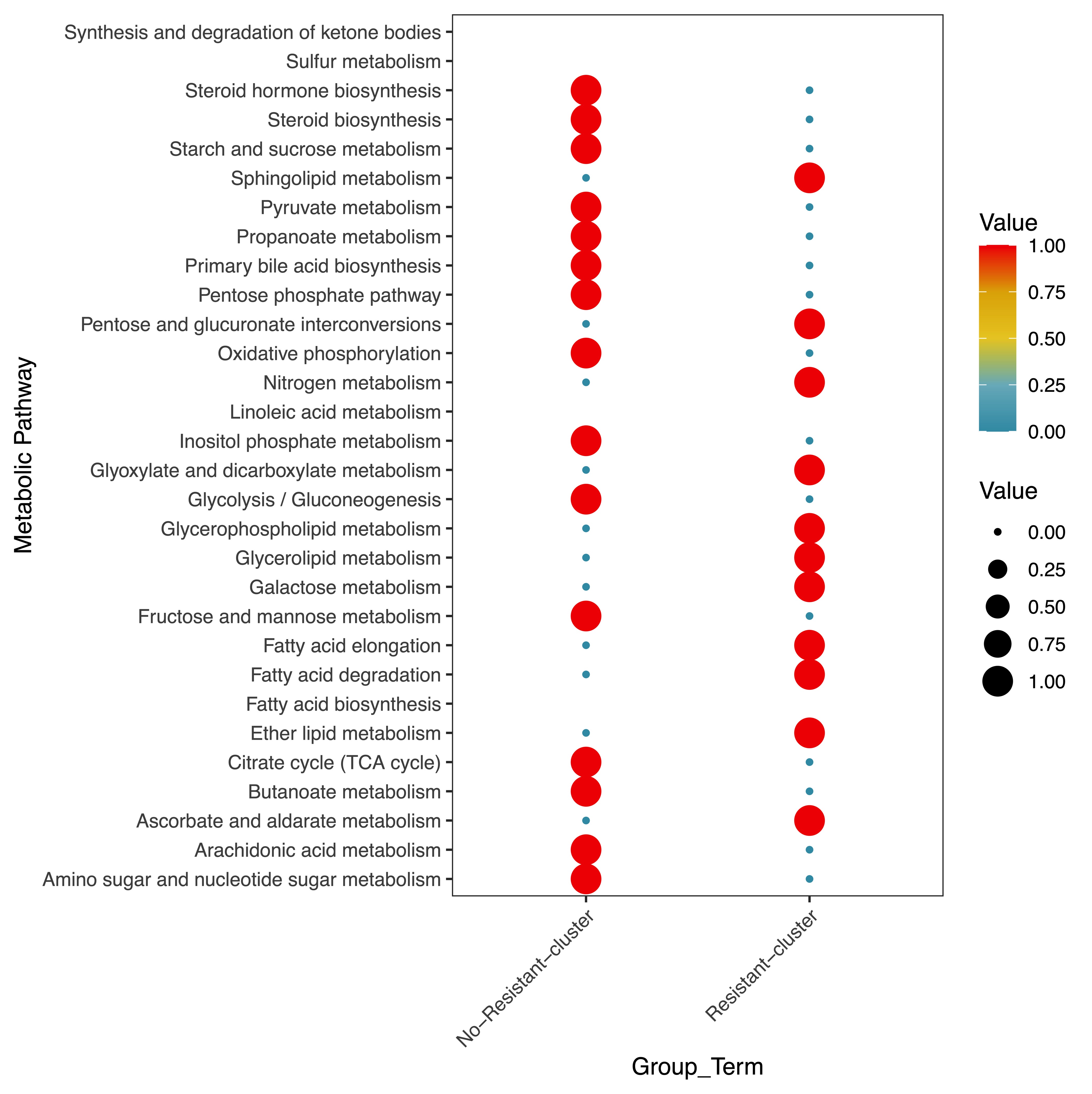

Supplement: Supplementary Figure 3 — Metabolism pathway score between resistance and non-resistance-subgroup. [file Image3.jpeg]
